# First year internal medicine residents’ self-report point-of-care ultrasound knowledge and skills: what (Little) difference three years make

**DOI:** 10.1186/s12909-021-02915-1

**Published:** 2021-09-07

**Authors:** Tanner Chahley, Ada W Lam, Samantha Halman, Kathryn Watson, Irene WY Ma

**Affiliations:** 1 Department of Medicine, University of Calgary Cumming School of Medicine, 3330 Hospital Dr NW, Alberta AB T2N 4N1 Calgary, Canada; 2 Department of Medicine, University of Alberta, Edmonton, Alberta Canada; 3 Department of Medicine, University of Ottawa, Ottawa, Ontario Canada; 4 Department of Community Health Sciences, University of Calgary, Calgary, Alberta Canada; 5 W21C, University of Calgary, Alberta Calgary, Canada

**Keywords:** Point-of-care ultrasound, Education needs assessment, Curriculum development, Internal medicine

## Abstract

**Background:**

With increasing availability of point-of-care ultrasound (POCUS) education in medical schools, it is unclear whether or not learning needs of junior medical residents have evolved over time.

**Methods:**

We invited all postgraduate year (PGY)-1 residents at three Canadian internal medicine residency training programs in 2019 to complete a survey previously completed by 47 Canadian Internal Medicine PGY-1 s in 2016. Using a five-point Likert scale, participants rated perceived applicability of POCUS to the practice of internal medicine and self-reported skills in 15 diagnostic POCUS applications and 9 procedures.

**Results:**

Of the 97 invited residents, 58 (60 %) completed the survey in 2019. Participants reported high applicability but low skills across all POCUS applications and procedures. The 2019 cohort reported higher skills in assessing pulmonary B lines than the 2016 cohort (2.3 ± SD 1.0 vs. 1.5 ± SD 0.7, adjusted *p*-value = 0.01). No other differences were noted.

**Conclusions:**

POCUS educational needs continue to be high in Canadian internal medicine learners. The results of this needs assessment study support ongoing inclusion of basic POCUS elements in the current internal medicine residency curriculum.

## Background

The use of point-of-care ultrasound (POCUS) in the assessment of medical patients is increasingly recognized. Support and endorsement statements of its use by several national and international internal medicine organizations importantly highlight the need for appropriate training [1–4]. In an attempt to better define an appropriate scope of use, the Canadian Internal Medicine Ultrasound (CIMUS) group identified four diagnostic applications (inferior vena cava, pulmonary B lines, pleural effusion, and abdominal free fluid) and three procedures (thoracentesis, paracentesis, and central venous catheterization) that should be included in the curriculum for core internal medicine residency programs [Postgraduate year (PGY)-1 to PGY-3] across Canada [5]. Foundational to these skills are multiple additional basic POCUS competencies such as knobology (knowledge and use of knobs and controls on the ultrasound device), ultrasound physics, Doppler, and safety principles [6]. To help optimize and monitor the delivery of high quality education, the CIMUS group also developed education indicators [7]. However, despite the presence of these guiding principles and documents, a consistent and significant barrier to POCUS curriculum development and implementation efforts has been the limited number of POCUS-trained faculty currently available [8]. In the face of limited available expertise, it may be preferable if faculty energy could be directed towards teaching a more focused and targeted curriculum.

In a 2014 national survey of Canadian medical schools, approximately half the schools had implemented POCUS education in their medical school curriculum [9]. Since that time, the ultrasound curriculum has been increasingly adopted worldwide [10, 11]. For example, in the United States, approximately one-third of medical schools had an ultrasound curriculum in 2014 [12]. By 2019, over 70 % reported having an ultrasound curriculum [13]. Although specifics within a medical student POCUS curriculum may vary, the basic foundational elements such as transducer selection, knobology, and safety principles of minimizing ultrasound dose delivery, such as the ALARA (as low as reasonably achievable) principle, are consistently agreed upon as critical curricular content [10, 14]. Therefore, if internal medicine residents are now entering residency with basic POCUS skills and knowledge learned at the medical school level, then educationally it may be more efficient to target POCUS training at a higher level. However, it is unclear whether or not residents starting in internal medicine residency have these basic skills. In our needs assessment survey in 2016, PGY-1 learners in internal medicine were largely novices, with minimal baseline POCUS knowledge and skills  [15]. These learning needs are similar to needs expressed by internal medicine learners in other countries, including the United States [16–19], the Netherlands [20], and Saudi Arabia [21, 22].

Given that much time has passed since our initial survey, we hypothesized that learners from more recent cohorts may be starting their residency training with more pre-existing POCUS knowledge and skills than previous cohorts. If more recent learners have higher knowledge and skills, we hope to then refine and update our curriculum accordingly.

## Methods

### Aim

This study seeks to compare the knowledge and skills of PGY-1 internal medicine residents across three Canadian centers with reported knowledge and skills from a similar cohort in 2016.

### Study Design

This multi-center cross-sectional survey study was conducted at three Canadian academic centers: University of Alberta, University of Calgary, and University of Ottawa. All three centers participated in the original 2016 study.

### Survey

We used the same survey that we previously developed and published, containing questions regarding learner opinions on 15 diagnostic applications, 9 procedures, and 18 knowledge items, in addition to questions on baseline demographic data [15]. Briefly, content validity of our survey was supported by literature review and expert input, and the draft survey was pretested on 8 non-internal medicine trainees for feedback on survey length, content, and clarity [15]. The final online survey (Survey Monkey Inc. San Mateo, California, USA; www.surveymonkey.com) was originally distributed to the trainees in 2016 and for this present study cohort, from August to October 2019. We sent up to two reminder emails between two and eight weeks to maximize participant response rate. No incentives were used in this study. No repeat pilot-testing of this survey was done for 2019. The results from the 2016 are previously reported [15] and only the data from the three centers included in the present 2019 study are used for comparison purposes. The fourth center was not able to participate in this present study due to competing workload demands precluding the requisite application to their ethics board.

### Participants

All postgraduate year (PGY) 1 internal medicine residents in 2019 at the three study institutions were invited to participate. Those who consented and completed the survey were included. Our survey results were then directly compared with the anonymized survey results for 47 PGY-1 learners who completed the identical survey at the same three institutions in 2016 [15].

### Outcomes

Perceived applicability of diagnostic applications and procedures to patient care in internal medicine was assessed on a 5-point Likert scale with 1 = not at all applicable, and 5 = very applicable. Self-reported level of skills/knowledge was also assessed on a 5-point Likert scale, with 1 = very poor, and 5 = very good. Skill gap was defined as the difference between perceived applicability of an application or procedure and self-rated skills/knowledge in that application or procedure [15].

 This study was approved by the University of Calgary Conjoint Health Research Ethics Board, the University of Alberta Research Ethics Board, and the Ottawa Health Science Network Research Ethics Board. Informed consent was obtained from all subjects. All subjects were over the age of 18. All methods were performed in accordance with the relevant guidelines and regulations.

### Statistical Analysis

Categorical variables between groups were compared with Fisher’s exact tests and chi-square tests. Continuous variables between groups were compared with Student’s two-sample t-tests. In comparing group differences in diagnostic applications and procedures, we applied Bonferroni corrections to p-values to control for the effect of multiple comparisons [23]. All analyses were performed using SAS version 9.2 (SAS Institute Inc., Cary, NC).

## Results

Of the 97 participants invited to the study, 58 completed the 2019 survey (response rate = 60 %). This response rate is similar to that in 2016, where 74 PGY-1 residents from the three institutions were invited and 47 completed the survey (64 %, *p* = 0.62). Baseline characteristics and procedural experience of the participants in both cohorts are outlined in Table 1. In general, compared to learners in the 2016 cohort, learners in 2019 reported lower prior procedural experience in ultrasound-guided paracentesis, thoracentesis, and central venous catheterization (Table 1). Both cohorts reported a similar degree of difficulty in finding procedural preceptors/supervisors (Table 1).

**Table 1:** Baseline characteristics, procedural experience, and stated experienced frequency of lacking procedural preceptors of postgraduate year (PGY)-1 participants in the 2016 and 2019 cohorts, with data presented as number (percentage %)

Variable	2016 PGY-1 cohort	2019 PGY-1 cohort	*p*-value
Total participants	*N* = 47	*N* = 58	0.62
Gender			
Male	24 (51 %)	32 (55 %)	0.84
Female	23 (49 %)	26 (45 %)	
**Number of ultrasound-guided paracentesis performed**			
None	5 (11 %)	16 (28 %)	0.02*
1–2	19 (40 %)	29 (50 %)	
2–5	16 (34 %)	9 (16 %)	
6–9	6 (13 %)	2 (3 %)	
10 or more	1 (2 %)	1 (2 %)	
**Number of ultrasound-guided thoracentesis performed**			
None	21 (45 %)	39 (67 %)	0.04*
1–2	19 (40 %)	15 (26 %)	
2–5	5 (11 %)	2 (3 %)	
6–9	0	0	
10 or more	1 (2 %)	0	
**Number of ultrasound-guided central line insertions**			
None	26 (55 %)	37 (64 %)	0.04*
1–2	10 (21 %)	18 (31 %)	
2–5	6 (13 %)	2 (3 %)	
6–9	3 (6 %)	0	
10 or more	1 (2 %)	0	
**Number of ultrasound-guided peripheral IV insertions**			
None	37 (79 %)	46 (79 %)	0.85
1–2	3 (6 %)	6 (10 %)	
2–5	3 (6 %)	2 (3 %)	
6–9	0	0	
10 or more	2 (4 %)	2 (3 %)	
**When has a lack of teacher/supervisor affected your ability to perform a procedure?**			
Never	2 (4 %)	2 (3 %)	0.88
A few times	4 (9 %)	8 (14 %)	
Many times	25 (53 %)	29 (50 %)	
Most of the time	12 (26 %)	16 (28 %)	

### Perceived applicability, self-reported skills, and skill gap in diagnostic applications

**Fig. 1:**
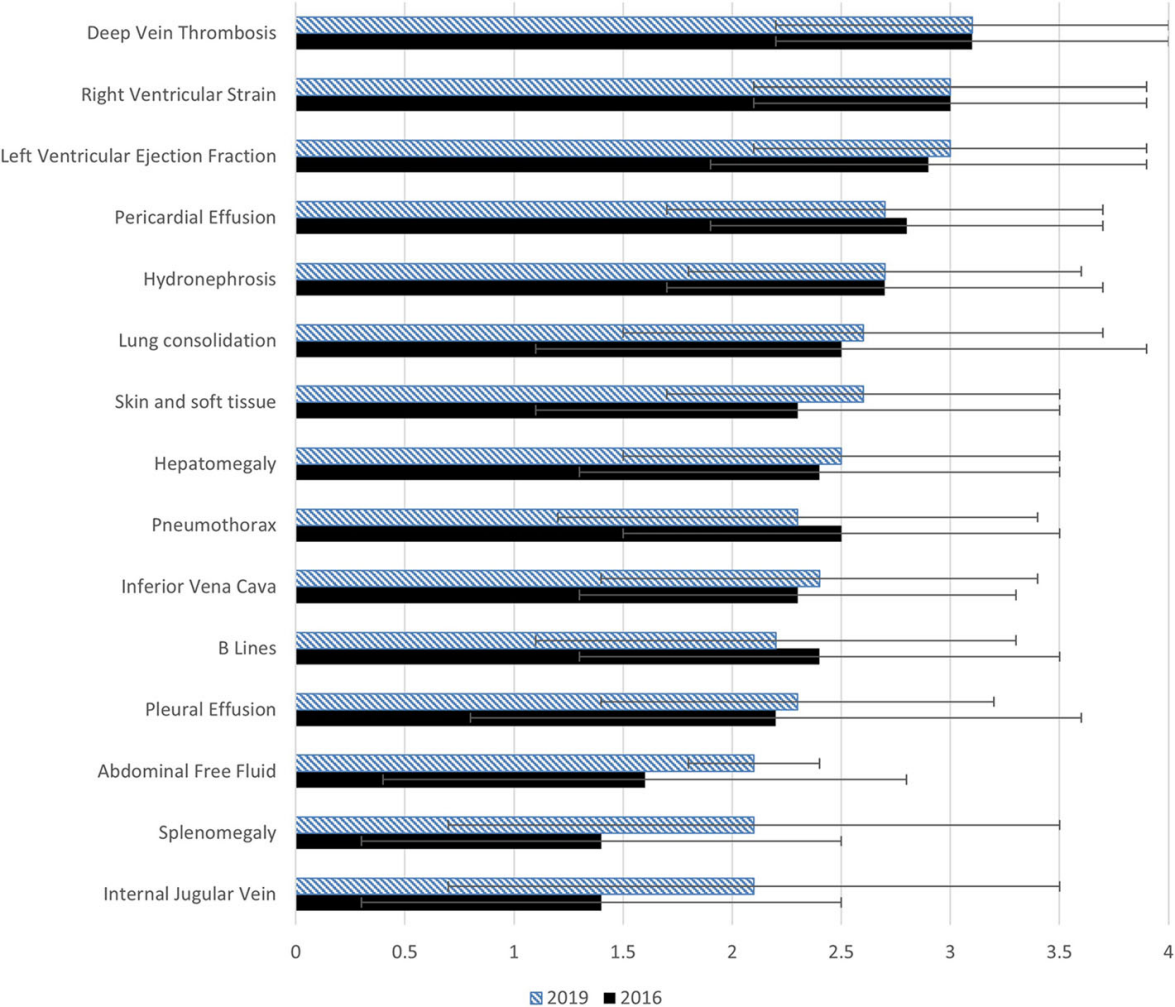
Skill gap of ultrasound diagnostic applications. Skill gap of ultrasound diagnostic applications is defined as the difference between perceived applicability of an application and self-rated skills, in the 2016 and 2019 cohorts, presented as mean gap; error bars indicate standard deviations

In both cohorts, ultrasound was perceived to be highly applicable to the practice of internal medicine in all diagnostic applications (Table 2). Learners continued to report low skills in all applications. No differences in learners’ perceptions were identified over time, except for the assessment of pulmonary B lines, where the 2019 cohort felt the application was more applicable to internal medicine than did the 2016 cohort [4.6 ± standard deviation (SD) 0.7 vs. 3.9 ± SD 1.0, adjusted p-value = 0.03]. The 2019 cohort also reported higher skills in assessing pulmonary B lines than the 2016 cohort (2.3 ± SD 1.0 vs. 1.5 ± SD 0.7, adjusted *p*-value = 0.01). Reported skill gaps were not significant in any other applications (Fig. 1). In both cohorts, skill gaps continued to be the highest in deep vein thrombosis scanning and cardiac applications (Fig. 1). Although the skill gap was higher in the 2019 cohort compared to the 2016 cohort for internal jugular vein, splenomegaly assessment, and abdominal free fluid, these differences were not statistically significant once adjusted for multiple comparisons.

**Table 2:** Perceived applicability, self-reported skill level, and skill gaps of diagnostic applications and procedures between 2016 and 2019 cohorts, with adjusted *p*-values. Data presented as mean score out of five and (standard deviation)

Diagnostic Application	2016 Applicability	2019 Applicability	Adjusted *p*-value	2016 Self-reported skills	2019 Self-reported skills	Adjusted *p*-value	2016 skill gap (SD)	2019 skill gap (SD)	Adjusted *p*-value
Internal jugular vein	4.4 (0.7)	4.6 (0.8)	1.00	3.0 (1.2)	2.5 (1.0)	1.00	1.4 (1.1)	2.1 (1.4)	0.99
Splenomegaly	4.4 (0.8)	4.4 (0.7)	1.00	2.2 (1.0)	1.8 (0.7)	1.00	1.4 (1.1)	2.1 (1.4)	0.96
Abdominal free fluid	4.8 (0.5)	5.0 (0.1)	1.00	3.3 (1.1)	2.9 (1.1)	1.00	1.6 (1.2)	2.1 (1.2)	1.00
Skin and soft tissue	4.5 (0.7)	4.3 (0.7)	1.00	2.2 (1.0)	1.7 (0.7)	1.00	2.3 (1.2)	2.6 (0.9)	1.00
Pneumothorax	4.6 (0.7)	4.4 (0.8)	1.00	2.1 (0.8)	2.1 (0.9)	1.00	2.5 (1.0)	2.3 (1.1)	1.00
B Lines	3.9 (1.0)	4.6 (0.7)	0.03*	1.5 (0.7)	2.3 (1.0)	0.01*	2.4 (1.1)	2.2 (1.1)	1.00
Internal vena cava	4.3 (0.9)	4.6 (0.6)	1.00	2.0 (0.9)	2.2 (0.9)	1.00	2.3 (1.0)	2.4 (1.0)	1.00
Pleural effusion	4.8 (0.5)	4.8 (0.4)	1.00	2.7 (1.1)	2.6 (0.9)	1.00	2.2 (1.4)	2.3 (0.9)	1.00
Lung consolidation	4.4 (1.0)	4.5 (0.9)	1.00	2.0 (0.9)	1.9 (0.8)	1.00	2.5 (1.4)	2.6 (1.1)	1.00
Hepatomegaly	4.5 (0.7)	4.3 (0.8)	1.00	2.2 (1.0)	1.8 (0.8)	1.00	2.4 (1.1)	2.5 (1.0)	1.00
Left ventricular systolic function	4.9 (0.4)	4.9 (0.4)	1.00	2.0 (0.9)	1.9 (0.9)	1.00	2.9 (1.0)	3.0 (0.9)	1.00
Pericardial effusion	4.8 (0.5)	4.8 (0.4)	1.00	2.1 (0.9)	2.2 (1.0)	1.00	2.8 (0.9)	2.7 (1.0)	1.00
Deep vein thrombosis	4.8 (0.5)	4.5 (0.7)	1.00	1.7 (0.8)	1.5 (0.7)	1.00	3.1 (0.9)	3.1 (0.9)	1.00
Hydronephrosis	4.5 (0.6)	4.5 (0.7)	1.00	1.9 (0.9)	1.8 (0.8)	1.00	2.7 (1.0)	2.7 (0.9)	1.00
Right ventricular strain	4.7 (0.5)	4.8 (0.5)	1.00	1.8 (0.8)	1.8 (0.8)	1.00	3.0 (0.9)	3.0 (0.9)	1.00
**Procedure**									
Paracentesis	4.9 (0.4)	5.0 (0.2)	1.00	3.4 (1.1)	2.8 (1.1)	1.00	1.6 (1.0)	2.2 (1.1)	1.00
Lumbar puncture	3.9 (1.0)	4.2 (0.9)	1.00	1.9 (1.0)	1.6 (0.7)	1.00	2.1 (1.3)	2.7 (0.9)	1.00
Central venous catheterization	4.9 (0.4)	4.8 (0.4)	1.00	2.6 (1.2)	2.1 (1.0)	1.00	2.4 (1.1)	2.7 (1.0)	1.00
Peripheral intravenous insertion	3.6 (1.1)	3.9 (0.9)	1.00	2.1 (1.0)	2.0 (0.7)	1.00	1.5 (1.5)	2.0 (1.1)	1.00
Joint aspiration	4.3 (0.8)	4.4 (0.7)	1.00	2.1 (0.9)	1.9 (0.8)	1.00	2.2 (1.3)	2.5 (1.0)	1.00
Arterial line insertion	4.5 (0.7)	4.6 (0.5)	1.00	2.1 (1.1)	2.0 (0.9)	1.00	2.4 (1.3)	2.6 (1.0)	1.00
Thoracentesis	4.9 (0.4)	5.0 (0.1)	1.00	2.3 (1.0)	2.2 (0.9)	1.00	2.6 (0.9)	2.8 (0.9)	1.00
Peripherally inserted central catheter	3.6 (1.3)	3.6 (1.1)	1.00	1.6 (0.9)	1.4 (0.5)	1.00	2.0 (1.3)	2.2 (1.1)	1.00
Incision and drainage	4.0 (0.8)	4.0 (0.9)	1.00	2.0 (0.9)	1.7 (0.7)	1.00	2.0 (1.1)	2.3 (1.1)	1.00

### Perceived applicability, self-reported skills, and skill gap in ultrasound-guided procedures

**Fig. 2:**
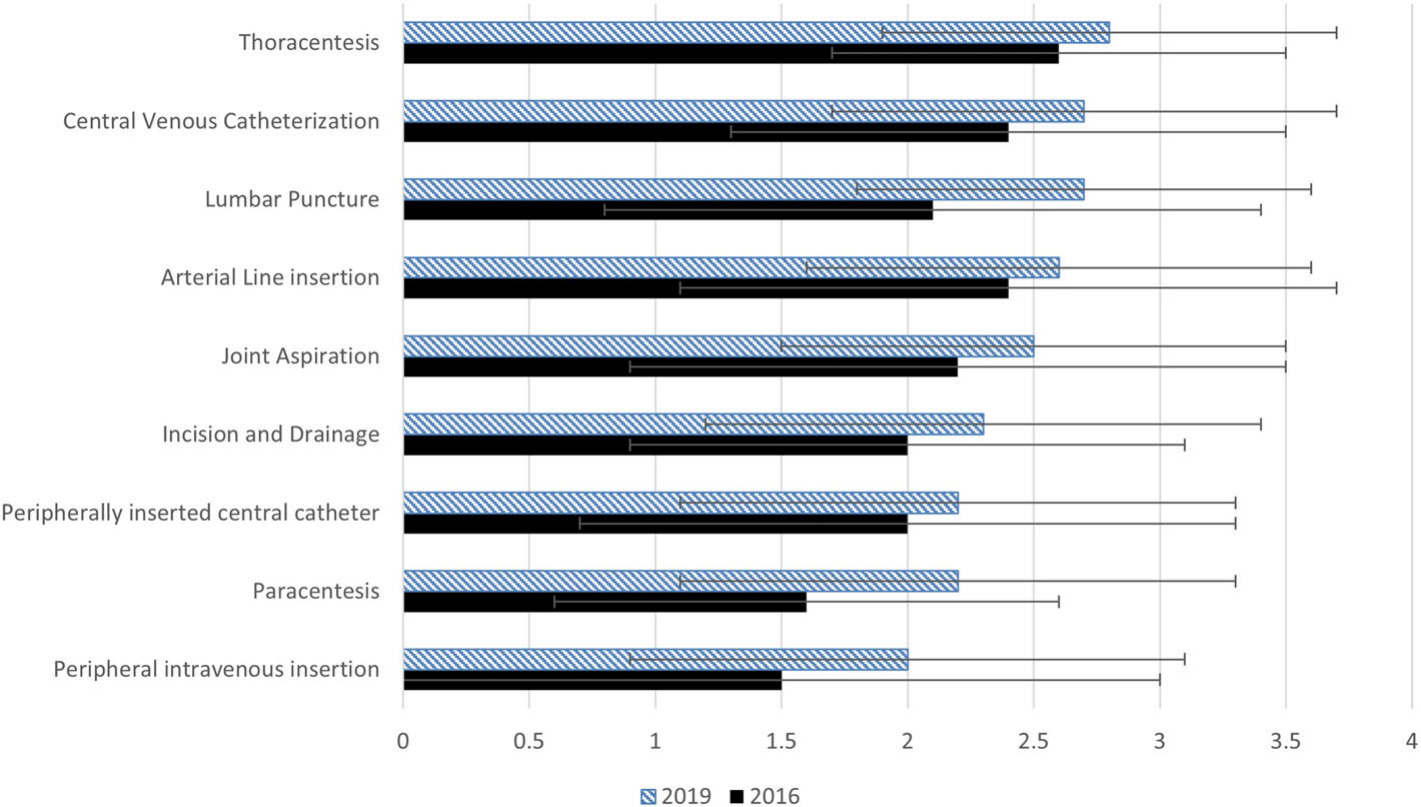
Skill gaps of ultrasound-guided bedside procedures. Skill gap of ultrasound-guided bedside procedures is defined as the difference between perceived applicability of a procedure and self-rated skills, in the 2016 and 2019 cohorts, presented as mean gap; error bars indicate standard deviations

Participants in both cohorts perceived that ultrasound-guided procedures were moderately-to highly applicable to the practice of internal medicine (Table 1). Both cohorts reported the highest applicability for paracentesis, central venous catheterization, and thoracentesis. Learners continued to report low skills in all ultrasound-guided procedures (Table 1). No significant differences in learners’ skill gaps were identified over time (Fig. 2). The largest perceived skill gap was reported in thoracentesis for both cohorts.

### Self-reported knowledge in ultrasound

Participants in both cohorts reported low knowledge on key ultrasound knowledge domains (Table 3), with a mean knowledge score of less than 3 for all items.

**Table 3:** Differences in self-reported knowledge on ultrasound domains between the 2016 and 2019 cohorts with adjusted p-values. Data presented as mean score out of five and (standard deviation)

	2016 Cohort	2019 Cohort	Adjusted *p*-value
The As Low as Reasonably Achievable (ALARA) Principle	1.9 (1.1)	1.8 (1.1)	1.00
B mode imaging	1.6 (0.8)	1.7 (0.8)	1.00
M mode imaging	1.6 (0.7)	1.7 (0.8)	1.00
Colour Doppler	1.9 (0.9)	1.8 (0.9)	1.00
Spectral Doppler - Pulsed wave	1.5 (0.9)	1.3 (0.5)	1.00
Spectral Doppler - Continuous wave	1.4 (0.6)	1.3 (0.5)	1.00
Power Doppler	1.4 (0.6)	1.3 (0.5)	1.00
Ultrasound artifacts	1.8 (0.8)	1.9 (1.0)	1.00
Transducer selection	2.5 (1.2)	2.6 (1.2)	1.00
Sterile transducer techniques	2.8 (1.3)	2.5 (1.2)	1.00
Knobology	1.7 (0.9)	2.0 (1.1)	1.00
Interpreting cardiac imaging findings	1.9 (0.9)	2.0 (0.8)	1.00
Interpreting lung imaging findings	1.9 (0.9)	2.2 (0.9)	1.00
Interpreting abdominal imaging findings	2.1 (0.9)	1.8 (0.9)	1.00
Interpreting renal imaging findings	1.8 (0.8)	1.7 (0.8)	1.00
Interpreting soft tissue imaging findings	1.7 (0.8)	1.6 (0.6)	1.00
Ability to discern when imaging is insufficient/inadequate	1.9 (1.0)	2.0 (0.9)	1.00
Ability to archive images or cine-loops	1.5 (0.6)	1.7 (0.9)	1.00

## Discussion

Our follow-up survey conducted three years after our initial needs assessment survey in 2016 continued to show a significant gap between perceived applicability and self-reported skill and knowledge in many ultrasound diagnostic applications and procedures for PGY-1 Internal Medicine residents at three Canadian centers. This gap was present even for basic foundational POCUS competencies such as basic imaging modes, knobology, and ultrasound safety principles (e.g. ALARA). With the exception of an increase in the perceived applicability of as well as self-reported skills in identifying pulmonary B lines in the 2019 cohort compared to the 2016 cohort, we found no significant differences otherwise noted over time. Specifically, junior learners continued to perceive ultrasound to be highly applicable to the practice of internal medicine, but upon entry into residency, continued to report limited skills across all aspects of POCUS.

Since 2016, concerted efforts had been made at improving internal medicine POCUS knowledge and skills across Canada, including published consensus-based curriculum and education indicators [5, 7]. Concurrently, ultrasound education has also increased at the medical school level in Canada [10, 11]. For example, the University of Calgary introduced ultrasound into physical examination and anatomy teaching in 2012 and 2014, respectively [24]. McGill University started its comprehensive longitudinal POCUS curriculum in 2013 and expanded its simulation-based POCUS training in 2017 [25]. The University of Saskatchewan, since 2018, has published on a number of their innovative medical student POCUS education initiatives [26, 27]. More recently the University of Ottawa published the results of on their pre-clerkship POCUS training [28]. With this increase incorporation of POCUS training in Canadian medical schools, we hypothesized that more recent cohorts of learners would enter residency with higher POCUS knowledge and skills. Contrary to our expectations, learners entering internal medicine residency not only continued to have low perceived POCUS knowledge and skills in applications specific to the practice of internal medicine, but also reported low skills in POCUS basics. Our findings argue against an attempt to eliminate basic POCUS concepts and skills from the internal medicine residency POCUS curriculum. These high learning POCUS needs in our participants are similar to those of other learners in internal medicine [16–22]. However, to our knowledge, there are currently no follow-up studies that evaluate changes in learning needs over time, thus it remains unknown whether the lack of improvement in reported baseline POCUS knowledge and skills is a finding unique to our three Canadian centers or that this finding is also present elsewhere.

Limitations of our study include the following: first, not all learners responded (response rate of 60 %). However, this response rate is comparable to, if not higher than, prior ultrasound needs assessment studies [16, 20, 29–31], and is in keeping with prior studies, where an overall response rate of 30–40 % were noted [32, 33]. Second, our results are based on self-reported learner measures, which are subject to bias and inaccuracies [34]. Learners may in fact know more (or less) than they report. However, even if our participants were under-estimating their self-reported skills and knowledge, the fact that learners *feel* their skills are limited mandates an educational response from the program, at least for skills where competencies are an expectation. Third, our survey distribution periods were not identical. Specifically, surveys distributed to in 2019 were earlier in the academic year (August – October) than for the 2016 cohort, where many of the surveys were completed towards the end of the learners’ academic year. Thus, the lower level of procedural experience reported by the participants in the 2019 cohort may be a reflection of less clinical exposure. Therefore, these differences should be interpreted with caution. Nonetheless, because the level of experience, knowledge, and skills of learners at the start of the training program, rather than at the end of the year, determines curriculum development efforts, our results are nonetheless helpful. Fourth, the lack of knowledge/skill increase over time may be a result of a number of factors, such as an insufficient or ineffective medical school curriculum, insufficient time for follow-up, or that the medical school POCUS curriculum is more targeted towards certain specialties, such as emergency medicine. While our study is unable to identify which factor(s) is/are responsible, it is highly probable that despite a general increase in medical school POCUS educational efforts, basic foundational concepts may not have been consistently or sufficiently taught. For example, at one site, knobology was noted to be a potential barrier to learning, despite its inclusion in the curriculum [24]. Future studies should evaluate not only the quantity, but the content and quality of POCUS education at the medical school level. Finally, our original 2016 survey had data from four training programs. Due to logistic reasons, this present study only captured data from three of the four sites. Generalizability of our study’s conclusions may therefore be limited.

## Conclusions

Our multi-center survey study shows that 2019 PGY-1 Canadian internal medicine residents rate diagnostic and procedural POCUS skills as highly applicable, but report low skill levels, at comparable levels to survey participants from an earlier cohort in 2016. Despite the perceived widespread integration of POCUS in medical schools, there continues to be a large POCUS skills and knowledge gap. Therefore, POCUS educators should not yet assume any significant learner baseline POCUS knowledge or skills when developing their internal medicine POCUS curriculum.

## Data Availability

The datasets generated and/or analyzed during the current study are not publicly available but are available from the corresponding author on reasonable request.

## Abbreviations

ALARA: As Low As Reasonably Achievable
CIMUS: Canadian Internal Medicine Ultrasound
POCUS: Point-of-care ultrasound
PGY: Postgraduate year
SD: Standard deviation
